# The feasibility of using a digital tool to enhance mobilisation following abdominal cancer surgery—a non-randomised controlled trial

**DOI:** 10.1186/s40814-023-01377-0

**Published:** 2023-08-23

**Authors:** Patrik Karlsson, Malin Nygren-Bonnier, Lars Henningsohn, Elisabeth Rydwik, Maria Hagströmer

**Affiliations:** 1 Department of Neurobiology, Division of Physiotherapy, Care Sciences and Society, Karolinska Institutet, Stockholm, Sweden; 2 Women’s Health and Allied Health Professionals Theme, Medical Unit Occupational Therapy & Physiotherapy, Karolinska University Hospital, Stockholm, Sweden; 3 Department of Clinical Science, Intervention and Technology, Division of Urology, Karolinska Institutet, Stockholm, Sweden; 4 Academic Primary Health Care Centre, Region Stockholm, Stockholm, Sweden

**Keywords:** ActivPAL, Oncology, Physiotherapy, System Usability Scale

## Abstract

**Background:**

Early mobilisation is strongly recommended following abdominal cancer surgery, but evidence on how to structure early mobilisation to improve outcomes and support patient adherence is lacking. Pedatim® (Phystec) is a novel digital tool designed to support mobilisation in hospital settings using prescribed activities and goals on a tablet. The aim of this study was to evaluate the feasibility of the Pedatim tablet to enhance mobilisation following abdominal cancer surgery.

**Methods:**

In a non-randomised feasibility trial design, participants were recruited between January and May 2022 at Karolinska University Hospital, Sweden. Participants used a Pedatim tablet from postoperative day 1 (POD 1) until hospital discharge. The primary objective was to evaluate process feasibility, regarding recruitment, compliance, and acceptability. Recruitment was measured by percentage of available patients included, eligibility criteria sufficiency, and number of dropouts. Compliance was measured by number of patients using versus not using the board. Acceptability was measured using the System Usability Scale. The secondary objective was to evaluate scientific feasibility, defined as an indication of treatment effects where physical activity was assessed using an activPAL accelerometer. Unforeseen events relating to the tablet were also registered.

**Results:**

Based on predetermined feasibility criteria, the overall study design was determined to be feasible regarding recruitment as 69% accepted participation (*n* = 20), compliance was 95%, and the acceptability mean score was high (77/100). Eligibility criteria were not feasible as 79% (*n* = 108) of available patients were excluded. The intervention was determined to be scientifically feasible, mean steps per day increased from 623 (SD 766) to 1823 (SD 1446), and mean sit-to-stand transitions per day increased from 11 (SD 8) to 29 (SD 12) POD 1–4. Technical issues emerged, highlighting the need for available technical support and “user champions” among healthcare professionals on the ward.

**Conclusions:**

Using the Pedatim tablet to enhance mobilisation following abdominal cancer surgery was deemed feasible, but a randomised controlled trial is needed to determine the tool’s effectiveness. The study process was determined to be feasible with revisions of the eligibility criteria needed before a future trial. Involving healthcare professionals and providing available technical support are important for future implementation.

## Key messages regarding feasibility

- What uncertainties existed regarding feasibility?
  As the Pedatim tablet is a novel digital tool, one of the main uncertainties regarding feasibility was whether or not the intervention was acceptable to the patients. It was also uncertain whether there are indications that the intervention is having a positive effect. Feasibility regarding key aspects of the study design was also of interest before a future trial, such as recruitment and compliance.
- What are the key feasibility findings?
  The overall study procedure, including the process feasibility of the study and the scientific feasibility of the tool, was deemed feasible. The tool was acceptable to the patients and promoted patient motivation and adherence to the mobilisation regime. Minor issues with eligibility criteria and practical issues with the tablet were identified which need to be addressed before a future study.
- What are the implications of the feasibility findings for the design of the main study?
  Before a future trial, minor adjustments to the screening and inclusion process are needed to include more eligible patients. The timeframe for screening and informing patients before hospital admittance needs to be longer (approximately 4–5 days in this study). Having a larger number of available tablets would also facilitate inclusion of patients in a future trial. A need for champion users of the Pedatim tablet among healthcare professionals and a need for readily available technical support emerged as important given that the tablet sometimes malfunctioned.

## Introduction

Surgery is a common treatment for solid cancer tumours, and finding measures to minimise postoperative complications for patients and shorten hospital stay, from an economic standpoint, is important [1]. Early mobilisation is regarded as a key component of enhanced recovery protocols and is thought to minimise postoperative complications such as respiratory insufficiencies, atelectasis, pneumonia, and venous thrombosis following abdominal surgery [2–5]. Increased time spent upright during the first postoperative days is also associated with a shorter length of stay (LOS) [6]. Early mobilisation is therefore strongly recommended following abdominal cancer surgery. However, evidence on how to structure and increase early mobilisation to improve outcomes and promote patient adherence is lacking [4, 5, 7, 8].

In a previous study, we evaluated the Activity Board (Phystec), a tool designed to support patient mobilisation in hospital settings, which is currently used in several postoperative wards at Karolinska University Hospital Sweden. The Activity Board is a whiteboard hanging on the wall of a patient’s room with prescribed activities and associated goals to support postoperative mobilisation, such as sitting, walking, and breathing exercises. Use of the Activity Board to enhance mobilisation has been shown to increase time spent upright and reduce time to first stool and flatus, as well as shorten length of stay in patients following abdominal cancer surgery [9]. User experience of the Activity Board has also been evaluated among both patients and healthcare professionals, indicating that the Activity Board enables participation and facilitates empowerment with rehabilitation for patients, and also enables healthcare professionals to support postoperative mobilisation in a structured and patient-centred manner [10, 11]. Even though the Activity Board is highlighted as a useful tool, the white board format has been described as obsolete, large, cumbersome, and demanding to clean, which prompts the need for a modern alternative [10, 11].

Pedatim® (Phystec) is a novel digital tool, previously only prototype-tested in clinical settings, which was developed by the same company as the Activity Board and is built on the same principals as the white board but displays this on a tablet, and is therefore a digital activity board. Even though Pedatim builds on the proven concept of the original Activity Board and the layout and design are similar, it is still a novel tool requiring evaluation. The digital layout might not be perceived as intuitive and might also be prone to technical issues and damage, further justifying the need for evaluation before use in clinical settings. Before an intervention is evaluated in a large-scale trial, it is preferable to determine first its feasibility and the feasibility of the potential study design [12, 13]. Therefore, the aim of this study was to evaluate the feasibility of the Pedatim tablet as a tool to enhance mobilisation following abdominal cancer surgery, with the primary objective of assessing the process feasibility of the study design, and the secondary objective of assessing the scientific feasibility of the tool itself, as proposed by Thabane et al. (2010) [12].

## Methods

### Study design

To determine if this novel digital tool could be useful to enhance mobilisation following abdominal cancer surgery in clinical settings, several aspects of the Pedatim tablet require evaluation. To evaluate these aspects of the Pedatim, a comprehensive randomised controlled trial is needed, but to successfully conduct such a trial in a complex clinical setting, rigorous preparation is key. Therefore, a feasibility design was chosen to gain information that could guide a future large-scale trial.

In this study, process feasibility is defined as recruitment, compliance, and acceptability of the intervention, as these are key elements required for the success and relevance of a future large-scale trial; scientific feasibility is defined as indication of treatment effects, and unforeseen events, to indicate whether or not the intervention has the desired effects on the patients, and if any aspects emerged regarding the intervention that needs to be addressed before a future trial. Based on our previous and ongoing research in supporting mobilisation and rehabilitation following abdominal cancer surgery in the clinical setting, it was not deemed necessary to gain experience of the randomisation process and identify potential dropout rates due to allocation to a control group before a large-scale trial. Therefore, a single-arm design was chosen.

This non-randomised feasibility study was conducted between January and May 2022 at the Karolinska University Hospital Solna, Sweden. The study was approved by the Swedish Ethical Review Authority, 2021–04323. Where applicable, this report follows the CONSORT statement for pilot and feasibility trials [14] and “Guidelines for reporting non-randomised pilot and feasibility studies” [15].

### Participants

The medical records of patients scheduled for abdominal cancer surgery (colorectal, ovarian, and bladder cancer) at the urology and gastrointestinal wards at the Karolinska University Hospital, Sweden, were screened to assess eligibility. Patients who were scheduled for abdominal cancer surgery with an expected postoperative hospital stay > 3 days were eligible for inclusion if over 18 years of age, they understood Swedish in speech and writing, and were able to walk (with or without aid). Patients were excluded if the surgery method or a complication during surgery resulted in mobilisation restrictions related to sitting and walking, if the patient had cognitive impairments, or if the patient participated in another clinical rehabilitation study. The last exclusion criterion was set due to several ongoing clinical rehabilitation studies at the time with conflicting protocols at the urology and gastrointestinal wards at the Karolinska University Hospital. The aim was to include an equal share of patients between the two wards.

Written information together with informed consent forms were then sent to patients fulfilling the inclusion criteria via mail or email. They were then contacted by telephone where the patient could ask questions about the study as well as be invited to participate by the project manager. Written consent forms were collected from patients at hospital admittance, usually the day before surgery.

Besides patients, physiotherapists, nurses, and assistant nurses working with the included patients were asked to participate in a survey regarding the usability of the tablet as part of the process feasibility evaluation. The inclusion of healthcare professionals was done using convenience sampling. At general assemblies on the wards, they were informed about the opportunity to leave their feedback anonymously regarding usability by filling in a form and leaving it in a mailbox.

### Intervention

**Fig. 1:**
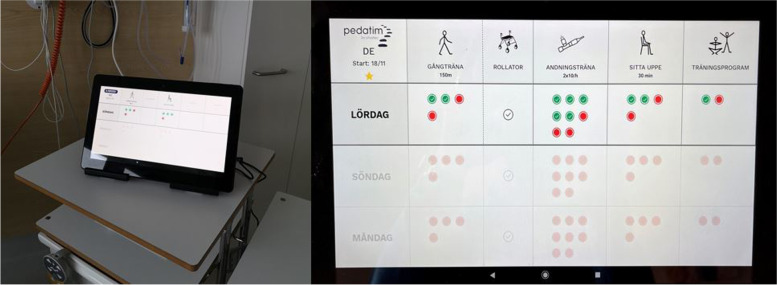
To the left, a picture of the Pedatim tablet in use is placed on a patient’s bedside table (by PK); to the right, a zoomed-in picture of the tablet with prescribed activities and goals (by Phystec AB)

Pedatim is a digital application with an activity board displayed on a tablet. It is designed by a Swedish private company (Scandinavian Phystec AB) to support mobilisation in hospital settings by illustrating activities and goals related to postoperative recovery. Activities such as sitting, walking, standing, and breathing exercises can be chosen, as well as goals regarding the frequency of these activities. The activities and goals are set by a physiotherapist or other healthcare professional on the ward together with the patient. The patient can then see the scheduled activities and goals on the tablet from day to day. A patient can scroll back and see previous days’ activities and goal fulfilment, planning of activities and goals can be set several days in advance, and subsequently, a patient can also scroll to see planned activities and goals for upcoming days. By checking boxes on the tablet (touching a red box on the screen), patients can indicate when an activity has been completed (the box then turns green). When a full set of activities has been completed, for example, all bouts of walking, confetti is displayed on the tablet as positive reinforcement, and when all activities are completed in a day, a gold star is awarded on the tablet as further positive reinforcement. An example of the Pedatim tablet in use can be seen in Fig. 1.

Prior to the start of this study, the research team received extensive training in the use of the tablet from the manufacturer, including both normal use and technical troubleshooting. The healthcare professionals working on the wards then received training from the research team in groups, focusing on set up of activities and goals, and navigating the patient interface. For the purposes of this study, five Pedatim tablets were leased from the manufacturer Phystec AB: four tablets to be used continually and one tablet as a backup in case of technical issues with one of the other tablets.

Upon returning to the ward following surgery and postoperative monitoring, which is usually on postoperative day (POD) one, a participating patient will receive the Pedatim tablet, along with information regarding its purpose and instructions on how to use it. A preliminary schedule with activities and goals is then set together with the patient. The patient interface of the tablet is then locked, and a patient can only see and interact with the scheduled activities and goals and see past achievements. The activities and goals can be revised at any time by a healthcare professional using a password. It is recommended that healthcare professionals revise the activities and goals regularly throughout a patient’s stay in accordance with the patient’s status to achieve progression and provide further support. Upon hospital discharge, the tablet is removed from the patient and reset before being given to the next patient. No information on the tablet is retained, nor collected.

### Outcomes

Evaluation of the process feasibility involves recruitment measured by the percentage of available patients included, sufficient eligibility criteria, and the number of dropouts, as well as compliance measured by the number of patients using the board versus not using the board and acceptability measured by the System Usability Scale (SUS) [16]. SUS is a standardised questionnaire for assessing the perceived usability of a system and has been validated for use with both general applications and digital health applications [16–18]. It consists of ten items with five Likert scale response options for each item; scoring is done using a matrix where each item response is converted to a number ranging from 0 to 4, and the sum of all items is then multiplied by 2.5; the result is a final score ranging from 0 to 100 [16]. The SUS score indicates whether system usability is high or low but does not give any information regarding user experience, such as why the usability might be perceived as high or low. Therefore, to gain an understanding of the user experience and potential barriers to using the Pedatim tablet, an open question was added along with the SUS: “Using your own words, describe your experience of using the Pedatim tablet”. As both patients and healthcare professionals are considered to be users of the Pedatim tablet, SUS will be collected from both patients and healthcare professionals.

Evaluation of the scientific feasibility of the intervention includes an indication of treatment effects which in this study is defined as the tablet’s potential to promote physical activity on the ward, measured by the number of steps per day, and sit-to-stand transitions per day using the activPAL3™ accelerometer during the patient’s stay on the ward. The activPAL3 is a small device placed on the front-centre of a patient’s thigh. It does not provide any feedback to the user, and the data are collected from the device after the measurement period has ended. It is validated to measure acceleration and position, which can be translated into steps, stepping speed, body posture, and posture transitions using PAL technology software PALbatch version 8.11.1.63 validation algorithm MORA v1.0, analysis algorithm VANE v0.1 with a wear time protocol of 24 h. The device is initiated from a set time point and can collect data for up to 14 days [19, 20]. LOS was measured using patients’ medical records, and unforeseen events were registered (such as technical or practical issues with the tablet).

#### Feasibility criteria

Criteria were defined to help determine the process feasibility. The criteria of an outcome must be met for the outcome to be determined as feasible, and if an outcome does not meet the criteria, a discussion must be had in the research group regarding the need for a potential modification for future studies [12]. The feasibility criteria for recruitment, dropout, and compliance are derived from experience from previous and ongoing studies on patients following abdominal cancer surgery [9, 21]. In these studies, we have been able to include approximately 50% of the available patients; the dropout rate has been 0–15%, and compliance is approximately 85%. The criteria for SUS are derived from the literature where a benchmark of a mean score of 68 has been established for both general applications and digital health applications; a mean score above 68 indicates good usability; and likewise, a mean score below 68 indicates lower usability [17]. Therefore, the following feasibility criteria were set for the respective outcome of the process feasibility in this study: recruitment > 50% of available patients, dropouts < 20%, compliance > 75%, and acceptability according to SUS score > 70.

### Data collection

During the first day on the ward following surgery, POD 1, a physiotherapist initiated the use of the Pedatim tablet together with the patient and applied the activPAL. Upon discharge from the hospital, the patient was asked to fill in the SUS and the open question; the Pedatim tablet and activPAL were then also removed from the patient, and the activPAL data were collected from the device. Demographic and medical data, as well as additional data on LOS, were collected through the medical records. The SUS aimed at healthcare professionals was available in the reception area beside a mailbox for the filled forms. Healthcare professionals working on the ward and using the tablet together with patients were asked to fill in the SUS form anonymously and leave it in the provided mailbox.

### Qualitative analysis

The open question delivered together with SUS was analysed using structured tabular thematic analysis [22]. The answers to the open question were imported into spreadsheet software (Microsoft Excel). Each answer was then read repeatedly, and initial notes were taken. Codes and preliminary themes were then formed for each answer. A table was then created with the text and preliminary themes to check agreement, frequency, and contrasts. The themes were then adjusted and properly named.

### Statistical analysis

Process feasibility was analysed in two steps: first with descriptive statistics using numerical values and percentages to describe included patients, dropouts, compliance, and eligibility criteria, and mean value to describe SUS. Then, second by determining whether or not the outcome was feasible (above or below the feasibility criteria level defined above). The continuous variables generated from the activPAL measurements, number of steps per day, and number of sit-to-stand transitions per day were analysed using descriptive statistics to evaluate participants’ physical activity patterns over the first few days following surgery. To analyse and illustrate the activPAL data, mean and standard deviation (SD) were calculated for the number of steps per day, and number of sit-to-stand transitions, and then presented using a box-plot to incorporate minimum and maximum values, median, outliers, and quartiles to illustrate physical activity levels as well as variation and progression over the first few days following surgery. As LOS varies, steps per day and sit-to-stand transitions were presented for the days with the most common denominator (POD 1–4).

## Results

**Fig. 2:**
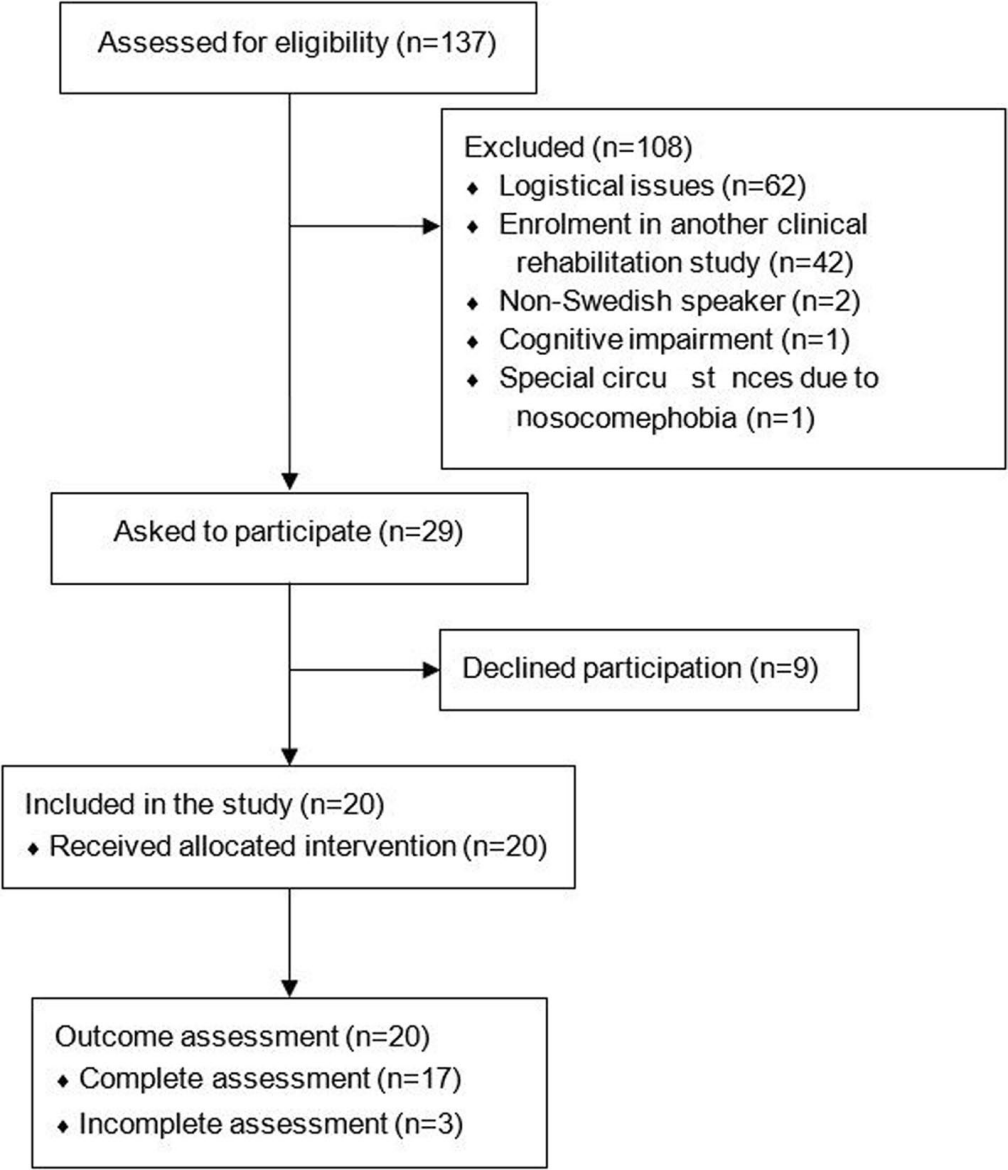
A flowchart describing the recruitment process and patient flow through the study

A total of 20 patients were recruited: eleven females and nine males, mean age 69 (min–max 41–81) years. Nine patients were recruited from the urology ward and eleven patients from the gastrointestinal ward. Included patients were scheduled for both open and laparoscopic surgery due to bladder cancer (*n* = 9), colorectal cancer (*n* = 8), and ovarian cancer (*n* = 3). A description of the participants can be seen in Table 1, and a flowchart of the recruitment process and patient flow through the study can be seen in Fig. 2.

**Table 1:** A description of the included participants’ characteristics

**Characteristics**	**Participants (*n* = 20)**
**Sex, *n* (%)**	
- Female	11 (55)
- Male	9 (45)
**Age (years)**	
- Mean (SD)	69 (11)
**Diagnosis, *n* (%)**	
- Bladder cancer	9 (45)
- Colorectal cancer	8 (40)
- Ovarian cancer	3 (15)
**Surgery method, *n* (%)**	
- Robot-assisted laparoscopic surgery	9 (45)
- Laparoscopic surgery	3 (15)
- Open surgery	8 (40)
**Length of stay (days)**	
- Mean (SD)	5.8 (1.4)
- Min–max	4–9

### Process feasibility

An overview of the process feasibility outcomes, feasibility criteria, and whether or not the criteria are met can be seen in Table 2.

**Table 2:** An overview of the results of the process feasibility outcomes and determination of feasibility

**Process feasibility domain**	**Result**	**Feasible**
Recruitment	- Of all available patients, only 21% were eligible for inclusion	No
	- Of patients asked to participate, 69% accepted	Yes (> 50%)
	- Dropouts were 0%	Yes (< 20%)
Compliance	- 95% of participants used the Pedatim tablet	Yes (> 75%)
Acceptability	- The mean score of the System Usability scale was 77	Yes (> 70)

#### Recruitment

A total of 137 patients were screened for eligibility; of these, 108 patients were excluded and 29 were eligible and asked to participate in the study; 20 patients accepted, and 9 patients declined. Subsequently, of all available patients screened, only 21% were eligible for inclusion, but 69% of patients who were asked to participate accepted. Therefore, the eligibility criteria were determined as not feasible. No participant dropped out of the study and the dropout rate was therefore determined to be feasible.

#### Compliance

Of the 20 participants who received the Pedatim tablet, one was unable to use the tablet due to technical issues. This resulted in a compliance rate of 95% which was therefore determined to be feasible.

#### Acceptability

A total of 17 patients and three healthcare professionals answered the SUS questionnaire resulting in a mean score of 77 out of 100. As previously mentioned, a mean score above 68 indicates good usability and, as per the feasibility threshold defined in this study, the acceptability of the intervention was deemed feasible.

The open question added to the SUS was answered by 14 participants: eleven patients and three healthcare professionals. The answers varied in length from a sentence to half a page of handwritten feedback, and the analysis of these answers resulted in three themes: “Easy to use and motivating”, “Support from healthcare professionals”, and “Technical support”. The Pedatim tablet was described as intuitive and easy to use, and the activities and goalsetting were seen as positive and encouraging. Patients described the need for support from healthcare professionals in the form of reassurance that they are using the tablet correctly, as well as to help revise their activities and goals as they progress. When faced with technical issues, patients described that the healthcare professionals on the ward often were unable to assist, prompting the need for technical support.

### Scientific feasibility

#### Indication of treatment effects

**Fig. 3:**
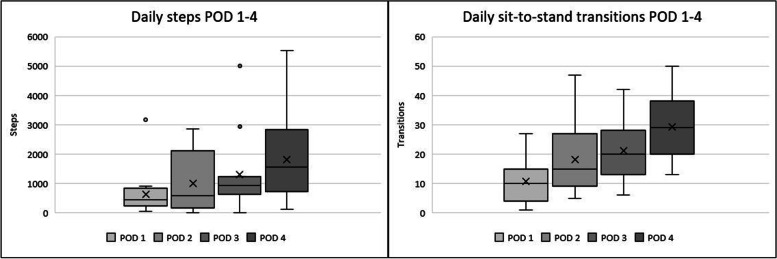
Box plot for daily steps and sit-to-stand transitions for postoperative day (POD) 1–4, including mean indicated by x (*n* = 15)

A total of 17 patients wore an activPAL from POD 1 to hospital discharge. LOS varied between 4 to 9 days, mean 5.8 days (SD 1.4), and median 5 days. Steps per day and sit-to-stand transitions are presented for measurements from POD 1 to POD 4 (*n* = 15) in Fig. 3. An overall progression in physical activity can be seen per day, but the individual variations are substantial. Mean steps per day increased from 623 (SD 766) POD 1 to 1823 (SD 1446) POD 4, and mean sit-to-stand transitions per day increased from 11 (SD 8) POD 1 to 29 (SD 12) POD 4.

#### Unforeseen events

During the study, a few technical issues emerged as the Pedatim tablet was merely a prototype at the time, and this was the first time it was tested in this clinical setting. Issues ranged from not being able to connect to the hospital network (mandatory in order for the tablet to function properly), to a few glitches and errors resulting in the tablets needing to be rebooted by a technical advisor from Phystec. All issues could be resolved by the technical advisor from Phystec and the technical support at the hospital. However, these issues periodically resulted in fewer available boards for patients to use and, subsequently, a slower rate of inclusion.

## Discussion

This study aimed to evaluate the feasibility of using the Pedatim tablet to enhance mobilisation following abdominal cancer surgery. This is the first time the tablet has been used and evaluated in this context, and thus, both the process feasibility of the study and the scientific feasibility of the intervention were of interest. Based on the predetermined feasibility criteria, the overall study design was determined to be feasible regarding recruitment, compliance, and acceptability of the tablet. However, the eligibility criteria were not deemed to be feasible and need to be addressed and revised before a potential future trial.

During the inclusion period, there were a total of 137 available patients scheduled for abdominal cancer surgery at the two participating wards, showing the potential for a large-scale trial. The number of available patients (approximately 150–200 patients per year) and inclusion rate of available patients (approximately 50%) is consistent with previous rehabilitation studies with similar patients and in similar contexts [9, 23, 24]. Due to two ongoing clinical rehabilitation trials with conflicting protocols at the participating wards, the number of eligible patients was greatly reduced. There were also some logistical issues: as the digital activity board was a prototype at the time, only four digital activity boards were available. This meant only four patients could be active in the study simultaneously, further reducing the possibility of including more patients. Furthermore, the screening was conducted approximately 1 week before admittance, which sometimes did not allow sufficient time to inform patients and receive consent before admittance. Subsequently, most patients were excluded because of these two external factors, greatly influencing the feasibility of the eligibility criteria, which is something that needs to be taken into consideration when planning a large-scale trial. These considerations include logistical issues, such as a larger number of tablets available, an earlier screening process in relation to hospital admittance, and recruiting patients without conflicting with other ongoing studies. The recruitment rate of the remaining available patients was satisfactory, and no patients dropped out, which indicates that the eligibility criteria might have been sufficient if not for external factors.

Of the 20 patients who received a Pedatim tablet, only one patient did not use the tablet due to technical issues with the device, indicating a high compliance overall. The result of the SUS also indicates a high usability as the mean score result of 77 is well above the previously stated benchmark of a mean score > 68 [17]. Together with the result of the open-ended question posed in conjunction with the SUS, both the compliance and acceptability of the intervention seem feasible and positive. This is somewhat also supported by previous studies conducted on Pedatim’s predecessor, the Activity Board, for which both patients’ and healthcare professionals’ experience of using the board has been evaluated, indicating that the concept of the board is easy to use and intuitive and that it promotes motivation and a patient-centred approach to postoperative mobilisation [10, 11]. However, a difference expressed by users of the Pedatim tablet in relation to users of the Activity Board is the need for technical support and education of healthcare professionals regarding the technical aspects of the tablet. As concluded from both the open-ended question and the unforeseen events regarding technical issues, supporting healthcare professionals’ technical literacy of the tablet and providing available technical support were crucial for the successful use of the Pedatim tablet in a clinical setting. This is further supported by the recommendations for implementation of e-health by Ross et al., in which training and education of all those involved are described as a key to success [25]. The LOS seen in this study was consistent with other studies conducted by our research team in similar patient groups, as well as with the literature [9, 26, 27].

An overall progression in physical activity among participants was observed. Physical activity levels and variations are consistent with what we have seen in a previous study of the same patient category and setting, using another method of supporting postoperative mobilisation [9]. In Porserud et al. (2019), patients receiving an intervention to enhance postoperative mobilisation walked an average of 1057 steps per day over the period of POD 1–3 and averaged 16 sit-to-stand transitions over the same period. However, the control group only walked an average of 360 steps per day and performed an average of 12 sit-to-stand transitions during the same period [9]. In another study assessing pre- and postoperative levels of physical activity using wearable monitors post elective abdominal surgery, a similar result could be seen in which patients walked an average of 1107 steps per day over the period of POD 1–3 [28]. This seems to support the scientific feasibility of the Pedatim tablet as a method to promote physical activity.

### Methodological considerations

A feasibility trial design was chosen to determine the feasibility of both the intervention and the study process. The recruitment protocol was based on researchers’ experience of conducting studies within this patient group, and the intervention also has a perceived value and potential benefits for these patients which might have contributed to the high inclusion rate of eligible patients, low dropout rate, and high compliance. There were however some recruitment issues, as described in the results, which not only influenced the feasibility of the eligibility criteria, but also prolonged the recruitment time in this study, ultimately reducing the number of patients who could be included within the study’s timeframe. The main arguments for not conducting a randomised feasibility trial including a control group were experience and knowledge gained from our previous studies focused on the same context and patient group, and we had earlier data on physical activity levels on the ward; in addition, we had a narrow timeframe. However, regardless of our previous experience, there are still limitations with this design as it could have provided us with data for the calculation of effect size, future power calculations, and a more accurate estimation of potential dropouts due to control group allocation. Acceptability of the intervention was translated into usability in this study, as the intervention mainly consisted of a novel digital tool and technical literacy that could impact the acceptability of using the tablet. In this feasibility study, we wanted to get a preliminary understanding regarding how the Pedatim tablet might be perceived by users, if it was at all feasible to expect frail older patients to use, and whether healthcare professionals perceived the tablet as easy to use or if more support might be needed. Thus, the aim was not to establish objectively the Pedatim tablet’s usability, but to gain a preliminary understanding of its usability and identify potential needs before conducting a large-scale trial. Therefore, SUS was used as it is the most used validated questionnaire for assessing the usability of technical products and eHealth applications [18, 29]. However, SUS only gives an indication of the usability and no information as to why the usability might be low or high, making it hard to understand how to proceed if the results are unfavourable. Furthermore, SUS is designed to give only a “quick and easy” way of assessing an application’s preliminary usability [16], giving no information to help with development or implementation. This is why the open-ended question was added in conjunction with the SUS form, which resulted in narratives that highlighted patients’ need for support from healthcare professionals and healthcare professionals’ need for available technical support. Despite numerous reminders during general assemblies on the wards, the number of healthcare professionals responding to SUS was low. The convenience sampling of healthcare professionals also contributes to a risk of sampling bias, especially in combination with the small sample. One could therefore argue that the healthcare professionals’ SUS responses should be eliminated from the analysis. However, as the healthcare professionals’ feedback provided contrast to the patients’ answers and addressed other aspects than the patients, their answers were included in the analysis. The SUS results from patients and healthcare professionals were analysed together, and we cannot know for certain how usability was perceived specifically among healthcare professionals as there were so few answers. The activPAL and LOS parameters were used to get an indication of the treatment effect of this intervention. Even though the activPAL is a validated device for measuring acceleration and position even among the frail and elderly, it has its limitations, such as problems with detecting low walking speeds which can lead to an underestimation of step counts [30, 31]. As this was not a randomised controlled trial and the number of participants in this study was limited, no certain conclusion can be drawn regarding the effect of the intervention. As mentioned, Pedatim is a novel tool designed by a Swedish private company that designs and sells products to support mobilisation in hospital settings. As such, the Pedatim tablet might not be widely available yet and the company stands to gain from its potential success, which may affect the external validity of this study. However, Pedatim is built on general principles to support behavioural change, and its format could be considered generic. Therefore, evaluating the Pedatim tablet as a tool to enhance mobilisation following abdominal cancer surgery is of clinical value, and some aspects of the tool might be generalisable.

## Conclusions

Using the novel digital tool, the Pedatim tablet, to enhance mobilisation to support mobilisation following abdominal cancer surgery was deemed feasible. However, a comprehensive randomised trial is needed to determine its effectiveness. The process of this study design was deemed feasible with revisions to the eligibility criteria required before a future large-scale trial. A progression of patients’ daily physical activity was observed when using the tablet and users express that it supports motivation. Involving healthcare professionals and providing available technical support are important for future implementation.

## Data Availability

Due to the nature of this research, participants in this study did not agree to their data being shared publicly, so supporting data are not available.

## Abbreviations

LOS: Length of stay
POD: Postoperative day
SUS: System Usability Scale
